# REPIC: a database for exploring the *N*^6^-methyladenosine methylome

**DOI:** 10.1186/s13059-020-02012-4

**Published:** 2020-04-28

**Authors:** Shun Liu, Allen Zhu, Chuan He, Mengjie Chen

**Affiliations:** 1 Section of Genetic Medicine, Department of Medicine, The University of Chicago, Chicago, IL 60637 USA; 2 Department of Chemistry and Institute for Biophysical Dynamics, The University of Chicago, Chicago, IL 60637 USA; 3 Howard Hughes Medical Institute, Chicago, IL 60637 USA; 4 Medical Scientist Training Program/Committee on Cancer Biology, The University of Chicago, Chicago, IL 60637 USA; 5 Department of Biochemistry and Molecular Biology, The University of Chicago, Chicago, IL 60637 USA; 6 Department of Human Genetics, The University of Chicago, Chicago, IL 60637 USA

**Keywords:** m^6^A modification, Database, Tissue specificity, Genome browser

## Abstract

The REPIC (**R**NA **EPI**transcriptome **C**ollection) database records about 10 million peaks called from publicly available m^6^A-seq and MeRIP-seq data using our unified pipeline. These data were collected from 672 samples of 49 studies, covering 61 cell lines or tissues in 11 organisms. REPIC allows users to query *N*^6^-methyladenosine (m^6^A) modification sites by specific cell lines or tissue types. In addition, it integrates m^6^A/MeRIP-seq data with 1418 histone ChIP-seq and 118 DNase-seq data tracks from the ENCODE project in a modern genome browser to present a comprehensive atlas of m^6^A methylation sites, histone modification sites, and chromatin accessibility regions. REPIC is accessible at https://repicmod.uchicago.edu/repic.

## Background

Over 150 chemical modifications have been identified in messenger RNAs (mRNAs) and non-coding RNAs (ncRNAs) [1]. Among them, *N*^6^-methyladenosine (m^6^A) is characterized as the most abundant and reversible mRNA internal modification [2, 3]. Numerous studies have emerged to establish m^6^A as a critical regulator of post-transcriptional gene expression programs which is involved with many cellular activities including splicing [4], translation efficiency [5], stability [6], export, and cytoplasmic localization [7] of m^6^A-modified mRNAs. Furthermore, m^6^A also impacts a series of physiological processes including, but not limited to, proliferation [8], development [9], neurogenesis [10], circadian rhythm [11], and embryonic stem cell differentiation [12].

With the advent of next-generation sequencing (NGS) technologies, several high-throughput sequencing methods (m^6^A-seq or MeRIP-seq [13, 14], PA-m^6^A-seq [15], m^6^A-LAIC-seq [16], miCLIP [17, 18], m^6^A-REF-seq [19], MAZTER-seq [20], and DART-seq [21]) have been developed to explore m^6^A modifications quantitatively across the entire transcriptome, paving the way for understanding their biological functions. These methods, especially m^6^A/MeRIP-seq, have been widely adopted to profile the m^6^A marks in a variety of cell lines and tissue types from multiple species. To better explore m^6^A data sets with increasing complexity, several databases (RMBase v2.0 [22], MeT-DB v2.0 [23], CVm6A [24]) and web servers (RNAmod [25], WHISTLE [26], SRAMP [27]) have been constructed to organize and integrate existing resources. Among these, RMBase v2.0 integrates information on sites of five or more types of RNA modifications, RBP binding sites, and single nucleotide polymorphisms, whereas MeT-DB v2.0 and CVm6A publish m^6^A peaks processed by their own pipelines from raw m^6^A sequencing data (Table 1). However, these databases have limitations. It has been shown that distinct m^6^A patterns occur in different developmental stages or tissue types, implying their dynamic regulation in a tissue-dependent manner [28]. Unfortunately, all of the above databases, except for CVm6A, simply combine m^6^A peaks across data sets without considering cell type or tissue specificity (Table 1). Furthermore, recent studies have uncovered associations between m^6^A modifications and promoters [29–31] or histone marks [32, 33], offering new insights into potential regulatory pathways and underlying mechanisms, through which m^6^A could influence transcriptional regulation and gene expression. However, to our knowledge, m^6^A modifications and epigenomic data have not been curated together well. New bioinformatic tools are needed for processing, analyzing, and visualizing the integration of these data.

**Table 1:** Summary of comparison between REPIC and published databases

Item	REPIC	RMBase v2.0*	MeT-DB v2.0	CVm6A
Species	11	13	7	2
Cell/tissue	61	45	40	31
Data set	49	39	26	23
Sample	672	524	437	130
Peak set	339	NA	185	69
De novo data processing	✓	✓**	✓	✓
Pipeline supported	✓	NA	NA	NA
Peak calling tools	exomePeak MeTPeak MACS2	exomePeak**	exomePeak	MeTPeak
Cell/tissue-based query	✓	NA	NA	✓
Genome browser	✓	✓	✓	✓
Intergenic m^6^A query	✓	NA	NA	NA
RNA modification type	m^6^A	5+***	m^6^A	m^6^A
Epigenomic data	1536	NA	NA	NA

*NA* not available

*Statistics from five modification types (m^1^A, m^5^C, m^6^A, Nm, and Ψ)

**Only m^6^A/MeRIP-seq and m^1^A-seq data were considered

***More than five RNA modification types

Here, we present the REPIC (**R**NA **EPI**transcriptome **C**ollection) database, which currently focuses on integrating m^6^A modifications with ENCODE epigenomic data (Table 1). The m^6^A modification peaks are generated by re-processing publicly available m^6^A-seq and MeRIP-seq data sets using a unified customized pipeline. REPIC allows users to query m^6^A modification sites by cell lines or tissue types with a user-friendly interface and provides a built-in genome browser for visualization. Overall, REPIC is a new resource designed to allow users to explore cell/tissue-specific m^6^A modifications and investigate potential interactions between m^6^A modifications and histone marks or chromatin accessibility.

## Construction and content

**Fig. 1:**
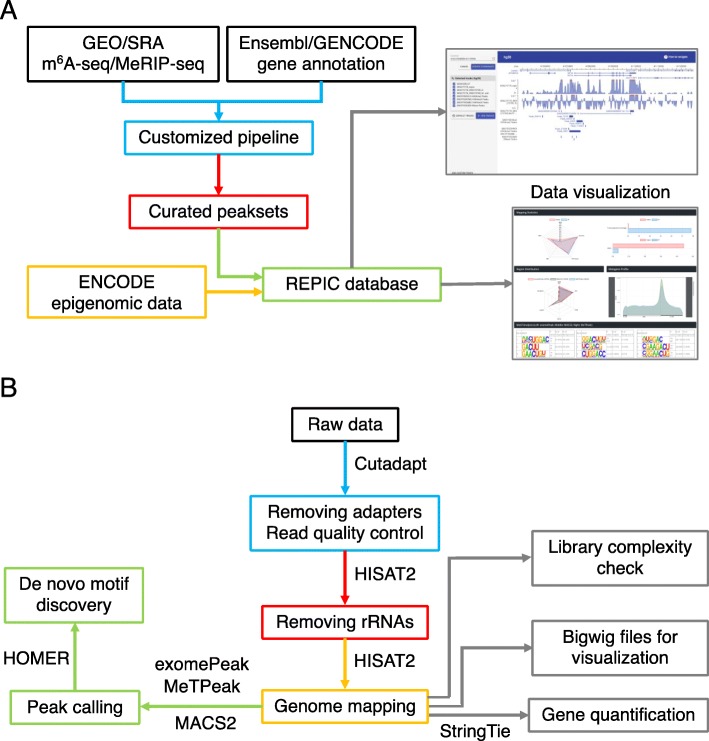
**a** Overall design of the REPIC database. **b** Schema of the customized pipeline for m^6^A-seq or MeRIP-seq data processing

The REPIC database collected m^6^A modifications and epigenomic sequencing data from different species. We designed a modern, user-friendly web portal for querying m^6^A modification sites and an interactive genome browser empowered by GIVE [34] for data visualization (Fig. 1a). The web application of the REPIC database was constructed using Apache v2.4.18, MySQL v5.7.25, and PHP v7.2.14. The data processing procedures starting from raw data sources are shown in Fig. 1b. To better disseminate the resource and facilitate downstream analysis, we provide curated data that can be downloaded from the REPIC database website.

### High-throughput sequencing data

Raw m^6^A-seq and MeRIP-seq data were manually collected through an extensive literature search and then retrieved from the Gene Expression Omnibus (GEO) and the Sequence Read Archive (SRA). In total, 607 m^6^A-seq and 544 MeRIP-seq run data were obtained from SRA. After merging different runs in the same experiment and excluding unpaired input-IP samples, 672 samples—which consisted of 339 pairs of input-IP data from 49 studies, covering 61 cell lines or tissue types in 11 organisms—were used for database construction (Additional file 1: Table S1). For epigenomic data, a total of 118 DNase-seq peak sets from 29 cell lines or tissue types, and 1418 histone ChIP-seq peak sets from 27 histone marks in 22 cell lines or tissue types in human and mouse, matching with curated m^6^A modification data, were downloaded from the ENCODE website (Additional file 1: Table S2 and S3).

### Genome annotation data

Human and mouse genome sequences and gene annotations were acquired from the UCSC Genome Browser [35] and GENCODE [36], respectively. *Arabidopsis thaliana* genome sequences and gene annotations were obtained from the Arabidopsis Information Resource (TAIR) [37]. The rest were downloaded from the Ensembl website [38]. The widespread versions of genome sequences and gene annotations for each of the 11 organisms were chosen for further analysis (Additional file 1: Table S4).

### Raw m^6^A-seq and MeRIP-seq data reprocessing

The aforementioned 339 pairs of input-IP data were re-processed by our customized pipeline [39, 40] (Fig. 1b). Briefly, adapters of raw sequencing data were clipped away by Cutadapt v1.15 [41]. Reads longer than 15 nt after trimming were first mapped to ribosomal RNAs (rRNAs) by HISAT2 v2.1.0 [42]. All unmapped reads were then aligned to genomes using HISAT2 v2.1.0 with default parameters. For samples with low mapping ratios, we used FastQ Screen [43] to find possible contaminants in those sample reads. To check library complexity, PCR duplicates were evaluated by MarkDuplicates from Picard v2.17.10 [44]. We then calculated the PCR duplicate proportion (PDP), which we defined as the number of PCR duplicate reads divided by the total number of mapped reads. Another three metrics, non-redundant fraction (NRF) and PCR bottlenecking coefficients 1 (PBC1) and 2 (PBC2), were quantified using ENCODE standards [45]. Input samples from m^6^A-seq and MeRIP-seq data were used to estimate gene expression levels by StringTie v1.3.4d [46]. If the library type was strand-specific, we further divided the sequence alignment data by strands. For visualization, log2 fold enrichment levels of m^6^A were calculated using bamCompare, and gene expression levels were reported in bins per million mapped reads (BPM) using bamCoverage from deepTools v3.0.2 [47]. exomePeak [48], MeTPeak [49], and MACS2 v2.1.1 [50] were used to detect peaks. For exomePeak and MeTPeak, parameters were set as follows: PEAK_CUTOFF_FDR = 0.05, WINDOW_WIDTH = 50, SLIDING_STEP = 10, MINIMAL_MAPQ = 20, FOLD_ENRICHMENT = 2, and REMOVE_LOCAL_TAG_ANOMALITIES=F. The values of the parameters FRAGMENT_LENGTH and READ_LENGTH varied under different library settings. Parameters in MACS2 were set as follows: -f BAM -B --SPMR --nomodel --keep-dup all. The values of the options -g, --tsize, and --extsize varied under different library settings. Finally, HOMER v4.9 [51] was used for motif enrichment analysis based on the top 2000 peaks ranked by their fold enrichment levels.

## Utility and discussion

### Evaluation of m^6^A-seq and MeRIP-seq data quality

**Fig. 2:**
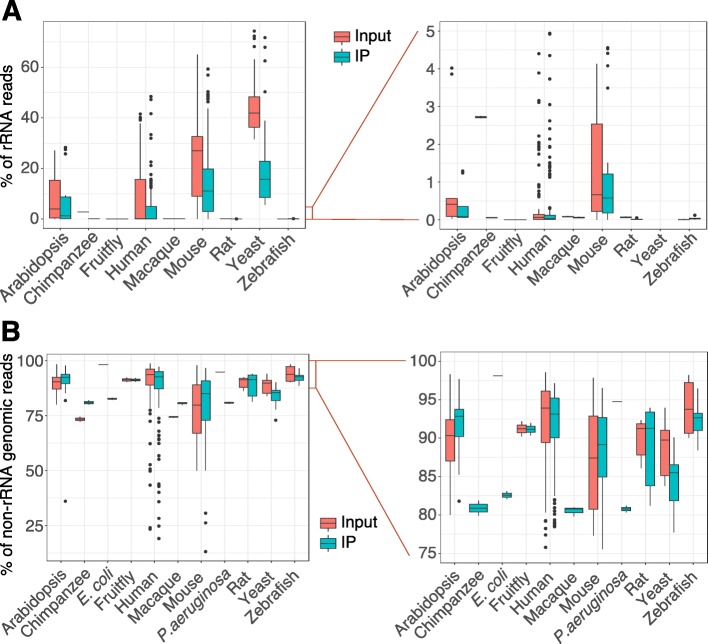
The quality of m^6^A-seq or MeRIP-seq reads mapping. Boxplots depicting the distribution of reads mapped to **a** rRNAs and **b** genomes in the input and IP samples, respectively. The *y*-axis in **a** and **b** represents the percentage of reads mapped to rRNAs and non-rRNA reads mapped to genomes, respectively. Both left-side panels show the whole range of the ratios and the right-side panels of **a** and **b** zoom in on the ranges of 0–5% and 75–100%, respectively

We applied our pipeline to re-process all collected m^6^A-seq and MeRIP-seq samples. As rRNAs could potentially interfere with mRNA expression quantification and peak calling, we first interrogated the rRNA content in each sample. rRNA reads comprised less than 30% of total reads in 566 samples (85.0% of the total), while 371 samples (55.7% of the total) contained a proportion of rRNA reads below 5% (Fig. 2a), suggesting that most samples were not subject to rRNA contamination. Next, we examined the counts of reads mapped to the genomes after filtering out rRNA reads. Five hundred seventy-one samples (85.7%) were shown to be of high quality with a genome mapping ratio greater than 75% (Fig. 2b). Sixteen human and 22 mouse samples with a low genome mapping ratio (< 60%) were detected as containing viral infection, vector or mycoplasma contamination, or other unknown conditions.

To further evaluate data quality, we assessed the library complexity of all samples by four metrics: PDP, NRF, PBC1, and PBC2, with the last three as defined by the ENCODE project [45]. The PDP values indicated that around 75% of the samples contained PCR duplicate proportions of greater than 50% (Additional file 2: Figure S1A), whereas the NRF values showed that only about 25% of the samples had a fraction of distinct, uniquely mapping reads greater than 50% (Additional file 2: Figure S1B). Both PDP and NRF values across the samples implied that multiple reads in the same positions of the genomes were prevalent. However, the decision of whether to remove them as PCR duplicates is an open question, since it is difficult to distinguish between artifacts of PCR amplification and real transcriptional events using current computational methods. Furthermore, direct removal of duplicate reads with the same mapping coordinates may introduce unwanted bias [52, 53]. Therefore, our pipeline keeps duplicated reads for downstream analysis. Unlike for PDP and NRF, about 90% of the input samples and 75% of the IP samples showed no severe (PBC1 > 0.5) or moderate (PBC2 > 3) levels of PCR bottlenecking (Additional file 2: Figure S1C and S1D) according to ENCODE standards. Overall, the two metrics PBC1 and PBC2 indicated that the library complexity of the majority of samples was of acceptable quality; thus, we considered them for further analysis. Nevertheless, we note that some characteristics of RNA biogenesis are more complicated than DNAs, so new metrics may need to be developed for the evaluation of RNA library complexity.

**Fig. 3:**
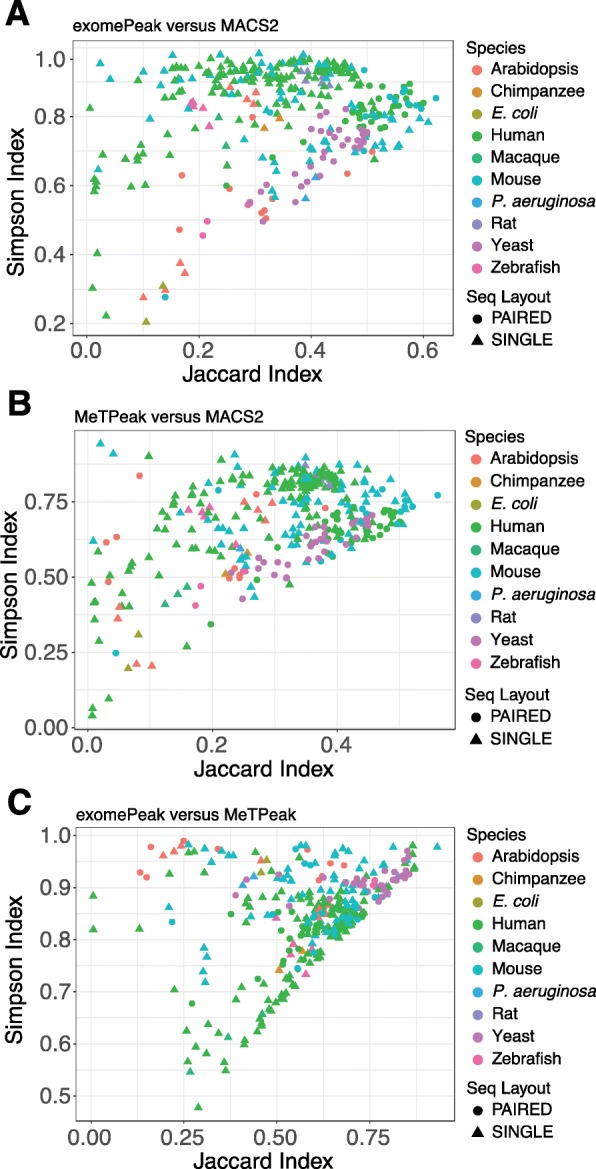
Evaluation of similarities of peak sets generated by three peak calling tools. Scatter plots showing the distributions of the Jaccard Index and Simpson Index from comparisons of **a** exomePeak versus MACS2, **b** MeTPeak versus MACS2, and **c** exomePeak versus MeTPeak across all samples. Paired-end and single-end sequencing types are represented by triangles and circles, respectively. Species are indicated by colors

Three peak calling tools—exomePeak, MeTPeak, and MACS2—have been widely used for m^6^A peak detection. exomePeak and MeTPeak were developed by the same group, but their algorithms vary. MeTPeak outperforms exomePeak based on robustness against data variance and detection of lowly enriched peaks [49]. However, with our processed data sets, exomePeak achieves better motif enrichment than MeTPeak. Unlike exomePeak and MeTPeak, both of which, by design, detect peaks across the transcriptome, MACS2 determines peaks genome wide. Thus, we can use MACS2 to obtain intronic and intergenic peaks. Because the algorithms of all three tools each have unique advantages, we applied them all to identify m^6^A peaks from the collected samples using fixed parameters. To assess the similarity of the peak sets identified by different tools, we adopted the Jaccard Index (JI) and Simpson Index (SI). JI is defined as the number of intersecting bases between two peak sets divided by the number of bases in the union of the two peak sets [54], and SI measures the ratio of the number of intersecting bases between two peak sets to the number of bases in the smaller of the two peak sets [55]. Thus, by definition, a given pair of peak sets has a higher SI than JI; the indexes have the same numerator, but the SI has a smaller denominator. To limit the comparisons at the transcriptome level, we considered only MACS2 peaks that overlapped with annotated transcripts. Unexpectedly, only about 13.6% and 3.0% of the peak sets from MACS2 had 50% or greater complete overlap (JI > 0.5) with those from exomePeak and MeTPeak, respectively (Fig. 3a, b). This observation indicated poor reproducibility between peak sets called by MACS2 and those by exomePeak or MeTPeak for the same given data sets. On the contrary, about 77.4% of the peak sets from exomePeak have JI > 0.5 when compared with those from MeTPeak (Fig. 3c). In addition, about 73.0% and 86.6% of the peak sets from exomePeak have SI > 0.75 with those from MACS2 and MeTPeak, respectively (Fig. 3a, c). However, the proportion of the peak sets between MACS2 and MeTPeak with the same SI was reduced to 37.7% (Fig. 3b). It suggests that peaks called by MACS2 and MeTPeak achieve lower consistency than those called by MACS2 and exomePeak. Taken together, exomePeak and MeTPeak agreed on over 75% of peak sets (JI > 0.5 or SI > 0.75), while MACS2 recovered limited peaks from exomePeak and especially MeTPeak.

### Cell- or tissue-specific m^6^A modifications

**Fig. 4:**
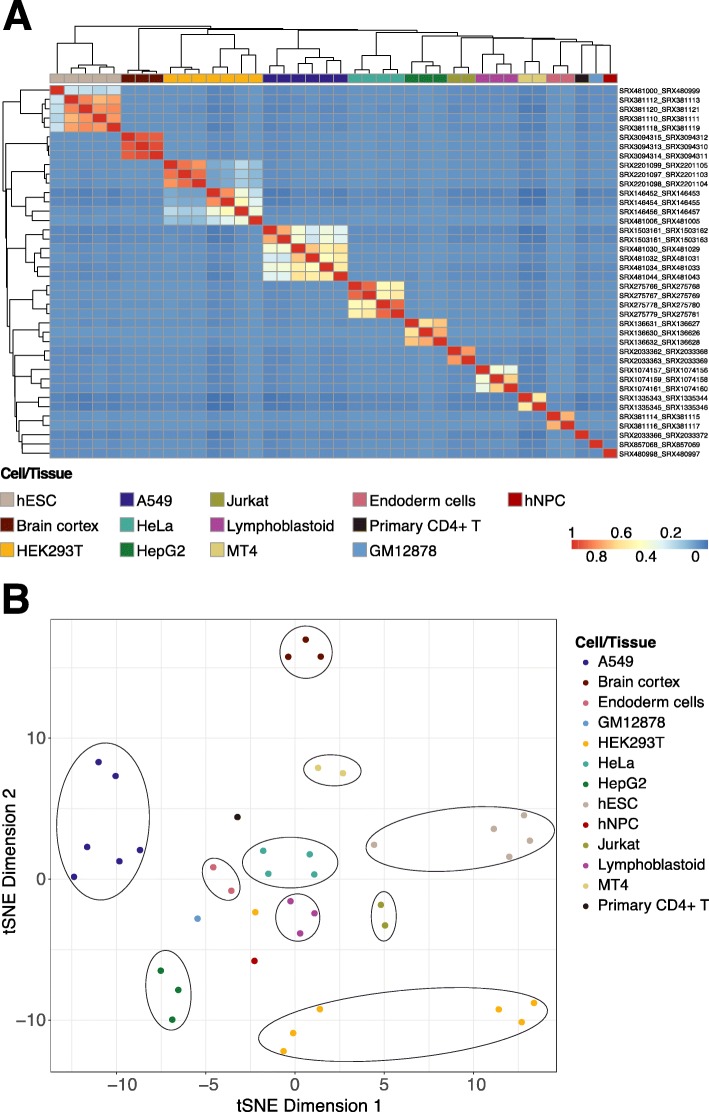
Cell- or tissue-specific m^6^A modifications. **a** Heatmap depicting the Pearson correlation of different human cell lines and tissues of the top 2000 genes ranked by CVs of fold enrichment levels of m^6^A peaks at stop codon regions (± 200 bp around the stop codons). The dendrogram was constructed using complete linkage based on Euclidean distances. Each row label represents the sample information in the format of “input_IP”. **b***t*-SNE plot displaying grouping patterns of different cell/tissue samples in a lower-dimensional space for the same data in **a**. Each dot represents a sample. Cell/tissue types are indicated by colors

As genes are expressed in a tissue-specific manner, we asked whether m^6^A modifications possess similar characteristics. According to the metagene profiles of m^6^A in mRNAs [56], we first considered five distinct genomic features: 5′ UTR, CDS, stop codon regions (± 200 bp around the stop codons), 3′ UTR, and whole regions. We then examined the top 2000 genes ranked by coefficients of variation (CV) of fold enrichment levels of m^6^A peaks at these regions across human cell lines and tissues. By comparing the m^6^A peak enrichment between samples at the 5′ UTR (Additional file 2: Figure S2A), CDS (Additional file 2: Figure S2B), 3′ UTR (Additional file 2: Figure S2C), and whole regions (Additional file 2: Figure S2D), we observed the strongest correlations among samples from the same cell lines or tissue types at stop codon regions (Fig. 4a), even when they were collected from different studies or labs. This phenomenon was also presented in the *t*-distributed stochastic neighbor embedding (t-SNE) [57] plot; samples from the same cell or tissue type were clustered together and clearly separated from other distinct groups (Fig. 4b). These results suggest that some highly dynamic m^6^A modifications at stop codon regions more so than those at other functional regions tend to be tightly controlled, perhaps in order to regulate cellular activities and processes in a cell line- or tissue type-specific manner, in response to different physiological stimuli or conditions.

**Fig. 5:**
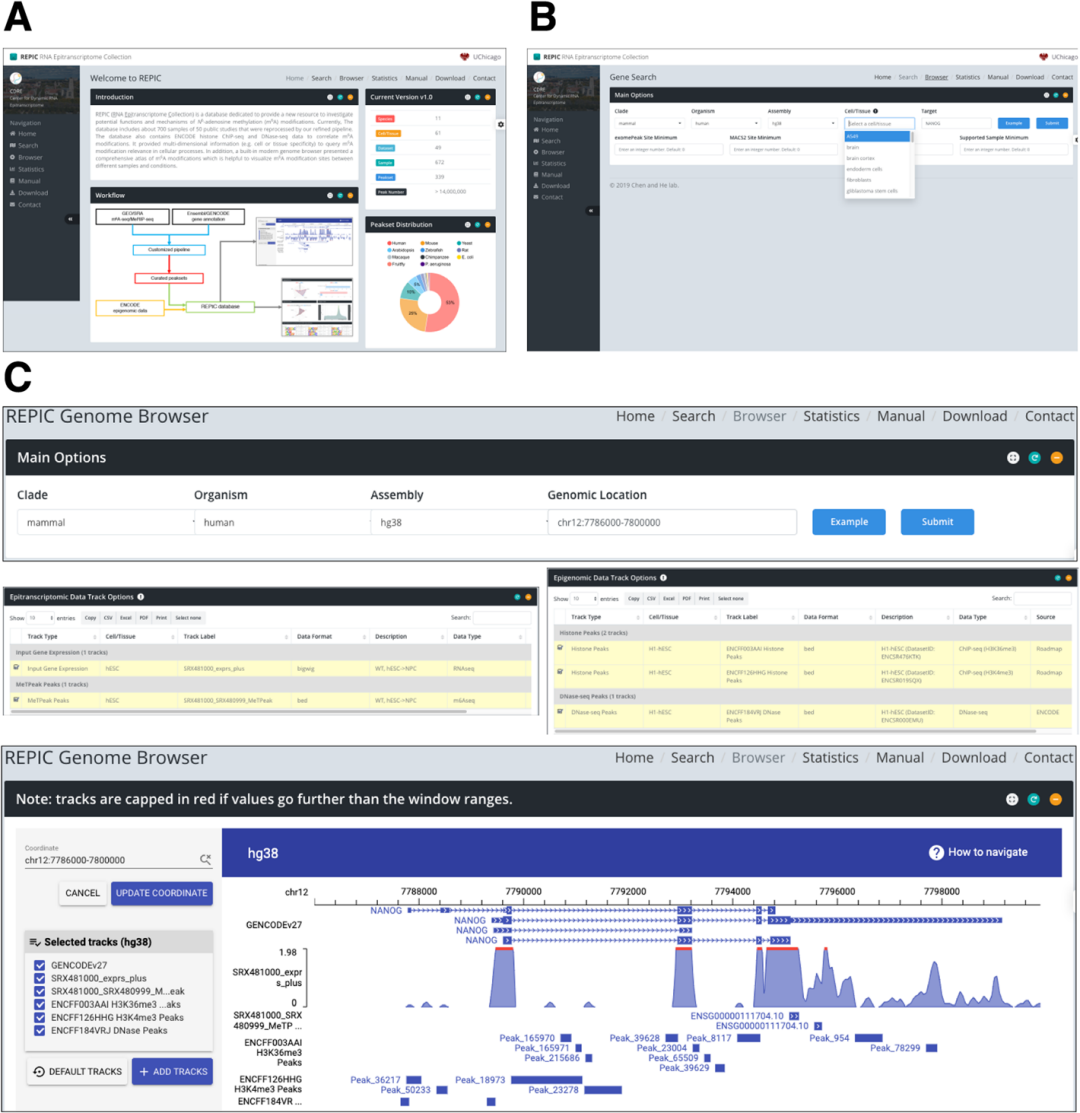
Screenshots of the web interfaces of the REPIC database. **a** The Home page. **b** The Search page. **c** Taking the query region near gene *NANOG* as an example, we show a visualization of m^6^A peaks, histone modifications, and chromatin accessibility in the genome browser

To offer insight into the cell line or tissue specificity of m^6^A modifications, REPIC supports the query of m^6^A modifications by cell lines or tissue types (Fig. 5a). On the Search page, we list options for all available cell lines and tissue types, next to filtering options that include the number of peak sites in the gene of interest and samples from which peaks were called (Fig. 5b). Once the submitted query is complete, a report will be presented in a user-friendly interface with the following information for each peak: genome position, other tools that identify an overlapping peak, fold enrichment, and genomic feature annotation (Additional file 2: Figure S3A). More sample information can be found in a separate window, including the data source, read mapping statistics, metagene profiles, and results from motif enrichment analysis (Additional file 2: Figure S3B).

### Visualization of m^6^A modifications and epigenomic data

The query on the Search page is limited to genes. To better display multi-dimensional m^6^A modification information across the entire genome, REPIC provides a genome browser empowered by GIVE to visualize m^6^A peaks, fold enrichment, and gene expression. As increasing evidence has shown that chromatin accessibility as well as epigenetic marks such as histone modifications defines the cell/tissue types [58, 59], we built REPIC to integrate DNase-seq and histone ChIP-seq data in order to investigate the possible correlations between these epigenomic characteristics and m^6^A modifications. As a result, a total of 3225 tracks comprising seven distinct track types (Additional file 1: Table S5) constitute the built-in genome browser. Like the UCSC Genome Browser or other similar genome browsers, a user can select multiple tracks to interactively display peak or expression profile data at a specific genomic location. In an example demonstrating the utility of the browser shown in Fig. 5c, we observe that H3K4me3 and DNase-seq peaks are located in the promoter region of the *NANOG* gene, indicating that it is actively transcribed in hESCs [12]. We also note that m^6^A modifications at the stop codon region are enriched with H3K36me3 peaks, which is consistent with the recently reported H3K36me3-dependent mechanism of m^6^A modification deposition [32].

### Future directions

As m^6^A modification detection technology has been applied to a variety of cell/tissue types with different conditions in distinct species, we will continue to collect new m^6^A/MeRIP-seq samples. In addition, with the increasing availability of transcriptome-wide sequencing data of m^6^A modifications at a single-nucleotide resolution as well as other RNA modifications including m^1^A, m^5^C, m^7^G, Ψ, and Nm, we will expand REPIC to catalog those as well. Another future development will be the integration of non-epitranscriptomic data such as RBP binding sites, GWAS, and GTEx data [60] to facilitate assessment and interpretation of RNA modifications.

## Conclusions

The current release of the REPIC database integrates millions of m^6^A peaks called by three popular tools from various cell/tissue types of multiple species. REPIC allows users to query m^6^A modification sites by specific cell lines or tissue types. Furthermore, hundreds of epigenomic data sets including chromatin accessibility and histone marks are included with the built-in genome browser to facilitate the interpretation of the functions of certain cell/tissue-specific m^6^A modifications, revealing their direct or indirect roles in influencing chromatin states and transcriptional regulation.

## Supplementary information

**Additional file 1: Table S1.** The list of sample information for the 339 input-IP paired samples. **Table S2.** The data set list of histone ChIP-seq peaks from ENCODE. **Table S3.** The data set list of DNase-seq peaks from ENCODE. **Table S4.** The genome assembly versions and gene annotation sources of 11 organisms. **Table S5.** The descriptions of tracks in the genome browser.

**Additional file 2: Figure S1.** Library complexity of m^6^A-seq or MeRIP-seq data. **Figure S2.** Correlation of m^6^A modifications in human cell lines and tissues categorized by genomic features. **Figure S3.** An example of the query of m^6^A modifications for a given gene.

**Additional file 3.** Review history.

## Data Availability

The lists of public m^6^A/MeRIP-seq, histone ChIP-seq, and DNase-seq data sets are also available in Additional file 1. Our customized pipeline is freely available on GitHub (https://github.com/shunliubio/easym6A) [39] and Zenodo (10.5281/zenodo.3742549) [40] under the GNU General Public License (GPL-v3.0). All 339 m^6^A peak sets can be downloaded from the REPIC data download center [61].
