# Supplementary material for: REPIC: a database for exploring the N6-methyladenosine methylome
Source: Genome Biol. 2020 Apr 28;21:100. doi: 10.1186/s13059-020-02012-4 (PMC7187508; doi:10.1186/s13059-020-02012-4)
Supplement: Supplementary file 2 — Additional file 2: Figure S1. Library complexity of m6A-seq or MeRIP-seq data. Figure S2. Correlation of m6A modifications in human cell lines and tissues categorized by genomic features. Figure S3. An example of the query of m6A modifications for a given gene. [file 13059_2020_2012_MOESM2_ESM.docx]

**Figure S1**. Library complexity of m^6^A-seq or MeRIP-seq data. Scatterplots illustrating the distribution of (a) PDP, (b) NRF, (c) PBC1, and (d) PBC2 values in input (x-axis) and IP (y-axis) samples. For each of these plots, the axes represent (a) the ratios of the number of PCR duplicate reads to the total number of mapped reads, (b) the number of distinct genomic locations covered by reads to the total number of genomic locations covered by reads, (c) the number of genomic locations covered by only one read to the number of distinct genomic locations covered by reads, and (d) the number of genomic locations covered by only one read to the number of genomic locations covered by only two reads, respectively. Triangles and circles represent paired-end and single-end sequencing data types, respectively. Color indicates species. All four types of values were calculated by our own custom scripts and then visualized by ggplot2.

**Figure S2.** Correlation of m^6^A modifications in human cell lines and tissues categorized by genomic features. The heatmap depicts the Pearson correlation of different human cell lines and tissues of the top 2,000 genes ranked by CVs of fold enrichment levels of m^6^A peaks at (a) 5’ UTR, (b) CDS, (c) 3’ UTR, and (d) whole regions of genes across cell lines and tissues. Cell/tissue samples were clustered by complete linkage, and the distances were measured by Euclidean distance.

**Figure S3**. An example of a query of m^6^A modifications for a given gene. Screenshots of the web interfaces are displayed to show practical use for (a) querying m^6^A modifications on a gene of interest (e.g., *NANOG*) from different cell lines or tissues, and (b) browsing information generated by data processing in associated samples.
