# Supplementary material for: REPIC: a database for exploring the N6-methyladenosine methylome
Source: Genome Biol. 2020 Apr 28;21:100. doi: 10.1186/s13059-020-02012-4 (PMC7187508; doi:10.1186/s13059-020-02012-4)
Supplement: Supplementary file 3 — Additional file 3. Review history. [file 13059_2020_2012_MOESM3_ESM.docx]

Review History

**First round of review**

**Reviewer 1**

**Have you tested the database?**

yes

**Are you able to assess all statistics in the manuscript, including the appropriateness of statistical tests used?**

yes

**Comments to author:**

Summary
In this paper, the authors describe a novel database for analysis and visualization of m6A mapping datasets. The database also incorporates histone modification and chromatin accessibility datasets form Encode, which can be used for comparative analyses. In addition, there are built-in tools for metagene analysis, motif identification, and overall distribution statistics and peak calling. Currently, there are only a small number of curated m6A databases which allow for user-specified searching of m6A sites in target RNAs. Therefore, this database is a welcome addition to the current repertoire of m6A analysis tools and offers several advantages over existing databases, including the inclusion of histone modification/DNA accessibility datasets and integration of data across multiple cell and tissue types. This online tool is likely to be of widespread use, although there are some points which should be addressed, as detailed below.

General comments:

1. It would be useful if the database's genome browser allowed for searching by gene name in addition to genome coordinates. Currently, I only see a way to search by gene name to see which datasets for a given species/cell/tissue type have overlapping peaks with that gene.

2. The authors mention several samples with mycoplasma or other contamination, but I could not find information on which samples this included. It would be helpful to list this information as part of the supplementary files so that users of the database can consider this information when analyzing datasets.

3. The popup that is generated when clicking on a particular dataset is small in size and cannot be expanded to include the full screen. It would be helpful if this could be expanded to full screen size.

4. "Non-Redundant Fraction (NRF), PCR Bottlenecking Coefficients 1 (PBC1) and 2 (PBC2), were measured according to the ENCODE standards." Please provide more details on what these standards include. At one point, a reference is provided, but the link given at this reference is dead.

5. On page 6: "rRNAs provide little information about transcriptome." It is unclear what is meant by this sentence—presumably the authors are trying to convey that rRNAs can interfere with analysis of mRNA m6A/expression levels. Please clarify this statement.

6. The database contains a nice user guide with instructions on how to search and use the various tools available. However, some of the figures in this user guide are small and hard to read. It may be helpful to enlarge these figures in the database to make them easier to visualize.

7. Fig 2A- Please specify what the rRNA ratio represents (% of rRNA reads relative to total reads?).

8. Fig 4A- The y-axis needs labels and it is unclear which studies are used for each row.

9. Fig S1- Please specify in the figure legend what the axes represent. The methods for generating these plots should also be included.

10. In the genome browser, the fold enrichment tracks are capped in red, presumably to indicate some threshold has been reached, but the meaning of this is not specified. Furthermore, details on how fold enrichment was calculated, as well as whether default or other parameters were used for the various peak-finding programs, are not given.

12. There are some minor grammatical errors throughout which should be corrected.

13. In the future, it would be good to include high-resolution m6A datasets (e.g., miCLIP, m6A-PAR-CLIP, etc.).

**Reviewer 2**

**Have you tested the database?**

yes

**Are you able to assess all statistics in the manuscript, including the appropriateness of statistical tests used?**

No

**Comments to author:**

In this manuscript, Shun Liu et al. described a database called REPIC recording m6A peaks from publicly available m6A-seq data using a unified pipeline. This database provided a fundamental resource for understanding the significance of m6A modification. However, I have several major concerns should be addressed before a further consideration.

1.     The authors should conduct more analyses to show the advantages of their database compared to the existing database such as RMBase and MeT-DB.
2.     Please confirm the pipeline "easym6A" is working. I have tried their pipeline, it does not work.
3.     Why did the authors select the three peak callers: exomePeak, MetPeak, and macs, but not select the methods used in the "Meyer et al. 2012, Cell". exomePeak and MetPeak were developed by the same group. MetPeak is the advanced version of exomePeak. Why not just use MetPeak? Moreover, do the authors consider combine the peaks from different peak callers?
4.      In page 9, line 4-8, the authors compared the m6As between different cell lines or tissues using m6A peak fold enrichment levels in stop codon regions. Could the authors explain the rationality of this? Why only choose stop codon regions?

**Authors Response**

**Point-by-point responses to the reviewers’ comments:**

*Response:*

*We thank both reviewers for their insightful comments and suggestions regarding our manuscript, and we appreciate their help for improving this manuscript. We performed additional analyses following the reviewers’ suggestions and integrated those additional results into the revised manuscript where appropriate. Below are our point by point responses to reviewers’ comments.*

Reviewer #1: In this paper, the authors describe a novel database for analysis and visualization of m6A mapping datasets. The database also incorporates histone modification and chromatin accessibility datasets form Encode, which can be used for comparative analyses. In addition, there are built-in tools for metagene analysis, motif identification, and overall distribution statistics and peak calling. Currently, there are only a small number of curated m6A databases which allow for user-specified searching of m6A sites in target RNAs. Therefore, this database is a welcome addition to the current repertoire of m6A analysis tools and offers several advantages over existing databases, including the inclusion of histone modification/DNA accessibility datasets and integration of data across multiple cell and tissue types. This online tool is likely to be of widespread use, although there are some points which should be addressed, as detailed below.

General comments:

It would be useful if the database's genome browser allowed for searching by gene name in addition to genome coordinates. Currently, I only see a way to search by gene name to see which datasets for a given species/cell/tissue type have overlapping peaks with that gene.

*We thank the reviewer for this great suggestion. Unfortunately, as the genome browser GIVE does not support searches by gene names, it is difficult to implement the suggested functionality within the framework that we have already adopted. However, in the browser index page (*[*https://repicmod.uchicago.edu/repic/browser.php*](https://repicmod.uchicago.edu/repic/browser.php),)*), we have made an effort to implement a function that enables users to search by gene name or gene ID in addition to genome coordinates. However, once users are directed to the interface of the genome browser, they can only browse the genome by genome coordinates.*

The authors mention several samples with mycoplasma or other contamination, but I could not find information on which samples this included. It would be helpful to list this information as part of the supplementary files so that users of the database can consider this information when analyzing datasets.

*We thank the reviewer for this great suggestion to provide more thorough detail on our samples. We added a field called “Remark” in Additional File 1 to list this information. We used FastQ Screen to search possible reasons for why some samples had low mapping ratios, and we added this description into the manuscript.*

The popup that is generated when clicking on a particular dataset is small in size and cannot be expanded to include the full screen. It would be helpful if this could be expanded to full screen size.

*We fully agree with the reviewer’s comments. We have expanded this popup component to full screen size.*

"Non-Redundant Fraction (NRF), PCR Bottlenecking Coefficients 1 (PBC1) and 2 (PBC2), were measured according to the ENCODE standards." Please provide more details on what these standards include. At one point, a reference is provided, but the link given at this reference is dead.

*We thank the reviewer for pointing this out. We provided formulas of these indexes in the website (*[*https://repicmod.uchicago.edu/repic/statistics.php*](https://repicmod.uchicago.edu/repic/statistics.php))*). Also, we have tested the link (*[*https://www.encodeproject.org/data-standards/terms/#library*](https://www.encodeproject.org/data-standards/terms/#library))*) of the reference again, and it is working now. This link can also be found in the database webpage (*[*https://repicmod.uchicago.edu/repic/statistics.php*](https://repicmod.uchicago.edu/repic/statistics.php))*). In addition, the definitions of these indexes are also referred in the legend of Fig S1.*

On page 6. "rRNAs provide little information about transcriptome." It is unclear what is meant by this sentence—presumably the authors are trying to convey that rRNAs can interfere with analysis of mRNA m6A/expression levels. Please clarify this statement.

*We thank the reviewer for pointing out this ambiguous statement. We excluded rRNAs because inclusion of rRNAs could potentially bring in unwanted noise to mRNA quantification and lead to inaccurate estimates. We have also clarified this in the manuscript.*

The database contains a nice user guide with instructions on how to search and use the various tools available. However, some of the figures in this user guide are small and hard to read. It may be helpful to enlarge these figures in the database to make them easier to visualize.

*We greatly appreciate the reviewer’s suggestion. We have implemented functionality that allows users to view a figure of larger size by clicking on it.*

Fig 2A- Please specify what the rRNA ratio represents (% of rRNA reads relative to total reads?).

*We have corrected the y-axis labels in the Fig. 2A and added details about the y-axis in the figure legend.*

Fig 4A- The y-axis needs labels and it is unclear which studies are used for each row. *We have labeled the y-axis in the Fig. 4A and described the labels in the figure legend.*

Fig S1- Please specify in the figure legend what the axes represent. The methods for generating these plots should also be included.

*We have added the definitions of the axis labels in the figure legend. They can also be referred to with this link (*[*https://www.encodeproject.org/data-standards/terms/#library*](https://www.encodeproject.org/data-standards/terms/#library))*). We have also added the description of the method of how these plots were generated.*

10. In the genome browser, the fold enrichment tracks are capped in red, presumably to indicate some threshold has been reached, but the meaning of this is not specified. Furthermore, details on how fold enrichment was calculated, as well as whether default or other parameters were used for the various peak-finding programs, are not given.

*We thank the reviewer for pointing out this lack of detail. We have added the description of red lines in tracks of the genome browser. In addition, we have included more details in the section “Raw m6A-seq and MeRIP-seq data reprocessing” in the revised manuscript, how fold enrichment was calculated, and which parameters in peak calling tools were used.*

There are some minor grammatical errors throughout which should be corrected.

*We thank the reviewer to point out these errors. We have carefully revised the manuscript, and hope the text reads more clearly now.*

In the future, it would be good to include high-resolution m6A datasets (e.g., miCLIP, m6A-PAR-CLIP, etc.).

*We thank the reviewer for this great suggestion. We are considering integrating these datasets in the next version of the database.*

Reviewer #2: In this manuscript, Shun Liu et al. described a database called REPIC recording m6A peaks from publicly available m6A-seq data using a unified pipeline. This database provided a fundamental resource for understanding the significance of m6A modification. However, I have several major concerns should be addressed before a further consideration.

The authors should conduct more analyses to show the advantages of their database compared to the existing database such as RMBase and MeT-DB.

*We fully agree with the reviewer’s comments. We compared REPIC with RMBase v2.0, MeT-DB v2.0 and CVm6A in Table 1 in the revised manuscript (also shown below). The table shows that 1) REPIC contains the largest number of samples, data sets, and cell lines or tissue types of m6A/MeRIP-seq data as well as annotated epigenomic features; 2) it supports the query of m6A modifications in intergenic regions; 3) it presents peak calling results from three different tools; 4) it provides a pipeline for users to analyze and visualize their own m6A/MeRIP-seq samples.*

**Table 1** Summary of comparison between REPIC and published databases.

| **Item** | **REPIC** | **RMBase v2.0*** | **MeT-DB v2.0** | **CVm6A** |
| --- | --- | --- | --- | --- |
| Species | 11 | 13 | 7 | 2 |
| Cell/Tissue | 61 | 45 | 40 | 31 |
| Data set | 49 | 39 | 26 | 23 |
| Sample | 672 | 524 | 437 | 130 |
| Peak set | 339 | NA | 185 | 69 |
| De novo data processing | V | V** | V | V |
| Pipeline supported | V  exomePeak | NA | NA | NA |
| Peak calling tools | MeTPeak | exomePeak** | exomePeak | MeTPeak |
|  | MACS2 |  |  |  |
| Cell/tissue-based query | V | NA | NA | V |
| Genome browser | V | V | V | V |
| Intergenic m^6^A query | V | NA | NA | NA |
| RNA modification type | m^6^A | 5+*** | m^6^A | m^6^A |
| Epigenomic data | 1536 | NA | NA | NA |

*: Statistics from five modification types (m^1^A, m^5^C, m^6^A, Nm and Ψ). **: Only m^6^A/MeRIP-seq and m^1^A-seq data were processed. ***: More than five RNA modification types. NA: Not available.

Please confirm the pipeline "easym6A" is working. I have tried their pipeline, it does not work.

*We thank the reviewer for pointing this out. We added more instructions about Installation in the* [*README.md*](http://README.md) *(*[*https://github.com/shunliubio/easym6A#installation*](https://github.com/shunliubio/easym6A#installation))*). In addition, we uploaded some test data for users to perform a rudimentary test run.*

Why did the authors select the three peak callers: exomePeak, MetPeak, and macs, but not select the methods used in the "Meyer et al. 2012, Cell". exomePeak and MetPeak were developed by the same group. MetPeak is the advanced version of exomePeak. Why not just use MetPeak? Moreover, do the authors consider combine the peaks from different peak callers?

*We thank the reviewer for pointing this out. We agree with the reviewer that we select the method used in "Meyer et al. 2012, Cell" in our pipeline. The core algorithm in the paper was Fisher’s exact test. We noticed that a number of studies used it for their m6A peak detection. We plan to add the results from this method in an upcoming future update. Although MeTPeak presented as more robust against data variance and missed fewer lowly enriched peaks than exomePeak, our exploratory analysis showed that the motif enrichment results from exomePeak are better than those from MeTPeak. As a result, in order to provide more information and options for users to explore their targets of interest, we included the peak calling results from both exomePeak and MeTPeak. Unlike exomePeak and MeTPeak, which are both designed for peak detection across the transcriptome, MACS2 determines peaks genome-wide. Thus, we can use MACS2 to obtain intronic or intergenic peaks. This is important because it can help us to analyze m6A methylation*

*in more RNA species, like nuclear or chromatin-associated RNA methylation[1, 2]. We also observed that a number of papers used MACS2 in their m6A methylation analysis. Therefore, we considered MACS2 as another peak caller in our pipeline. We noticed that some peaks identified from MACS2 can span exon-intron junctions. They may originate from the algorithm that MACS2 extends reads in the 5’-to-3’ direction across the genome instead of the transcriptome or real transcript events that current annotations fail to include. Thus, it is not plausible to merge or intersect these peaks with those from exomePeak and MeTPeak, as it may eliminate the advantages between different algorithms or underestimate the complexity of the transcriptome. However, we have marked which peaks from one peak caller overlap with those from other peak callers. The information is helpful as it can indicate which peaks may be of higher confidence. It has been and is available on the REPIC website.*

In page 9, line 4-8, the authors compared the m6As between different cell lines or tissues using m6A peak fold enrichment levels in stop codon regions. Could the authors explain the rationality of this? Why only choose stop codon regions?

*We thank the reviewer’s comments for us to clarify our approach. We performed the comparison analysis across cell lines using m6A peak fold enrichment levels at the 5’ UTR, CDS, stop codon regions, 3’ UTR, and whole genic regions, respectively. We found that the results with stop codon regions presented the strongest within-cell-type correlations and best separation for different cell types. This suggests that some high-CV or highly dynamic m6A modifications at stop codon regions tend to carry out cell line-specific functions more so than those at other functional regions. We have added a discussion on this in the revised manuscript. Heatmaps for the 5’ UTR, CDS, 3’ UTR and whole regions have been added as well in Figure S2 (also shown below).*

**A	5’ UTR	B	CDS**

1

0.8

0.6

0.4

0.2

0

1

0.8

0.6

0.4

0.2

0

**C	3’ UTR	D	Whole regions**

1

0.8

0.6

0.4

0.2

0

0.8

0.6

0.4

0.2

0

1

**Cell/Tissue**

hESC	A549	Jurkat	Endoderm cells	hNPC

Lymphoblastoid	Primary CD4+ T

Brain cortex

HeLa

|  | HEK293T HepG2 MT4 GM12878 |
| --- | --- |

*Figure S2. Correlation of m6A modifications in human cell lines and tissues categorized by genomic features. The heatmap depicts the Pearson correlation of different human cell lines and tissues of the top 2,000 genes ranked by CVs of fold enrichment levels of m6A peaks at (a) 5’ UTR, (b) CDS, (c) 3’ UTR, and (d) whole regions of genes across cell lines and tissues. Cell/tissue samples were clustered by complete linkage, and the distances were measured by Euclidean distance.*

*Bertero A, Brown S, Madrigal P, Osnato A, Ortmann D, Yiangou L, Kadiwala J, Hubner NC, de Los Mozos IR, Sadee C, et al: The SMAD2/3 interactome reveals that TGFbeta controls m(6)A mRNA methylation in pluripotency. Nature 2018, 555:256-259.*

*Liu J, Dou X, Chen C, Chen C, Liu C, Xu MM, Zhao S, Shen B, Gao Y, Han D, He C: N (6)-methyladenosine of chromosome-associated regulatory RNA regulates chromatin state and transcription. Science 2020, 367:580-586.*

**Second round of review**

**Reviewer 1**

The authors have addressed each of my concerns and I think that this improved database will be helpful to the research community.

**Reviewer 2**

All my questions have been addressed. I have no further comment.
